# Poly[di­aqua­[μ-1,4-bis­(pyridin-4-ylmeth­yl)pip­era­zine][μ-4-(carboxyl­atoeth­yl)benzoato]nickel(II)]

**DOI:** 10.1107/S2414314623007885

**Published:** 2023-09-22

**Authors:** Gabrielle J. Gaskin, Robert L. LaDuca

**Affiliations:** a E-35 Holmes Hall, Michigan State University, Lyman Briggs College, 919 E. Shaw Lane, East Lansing, MI 48825, USA; Vienna University of Technology, Austria

**Keywords:** crystal structure, nickel, coordination polymer, diamondoid network.

## Abstract

The title compound, [Ni(C_10_H_8_O_4_)(C_16_H_20_N_4_)(H_2_O)_2_]_*n*_, contains Ni^II^ cations octa­hedrally surrounded within an [O_4_N_2_] coordination set. The cations are linked by 4-(carb­oxy­eth­yl)benzoate (ceb) and 1,4-bis­(pyridin-4-ylmeth­yl)piperazine (bpmp) ligands into tri-periodic diamondoid (**dia**) networks. The fivefold inter­penetrated **dia** networks are held into the crystal structure by means of O—H⋯O hydrogen bonding between bound water mol­ecules and unligated carboxyl­ate O atoms of the ceb ligands.

## Abstract 2

The divalent nickel compound {[Ni(ceb)(bpmp)(H_2_O)_2_]_*n*_, (ceb is (4-(carb­oxy­eth­yl)benzoate; bpmp is 1,4-bis­(pyridin-4-ylmeth­yl)piperazine) crystallizes as a fivefold inter­penetrated tri-periodic coordination polymer without co-crystallized species.

## Graphical abstract

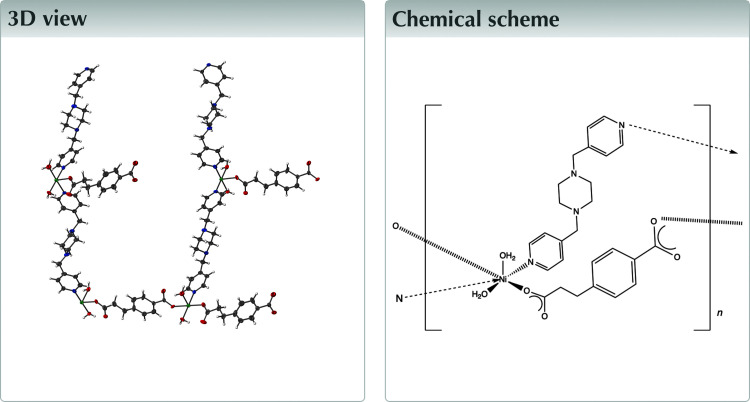

## Structure description

Our group has demonstrated the utility of 1,4-bis­(pyridin-4-ylmeth­yl)piperazine (bpmp) for the construction of divalent metal coordination polymers with a remarkable variety of inter­esting topologies (Robinson *et al.*, 2015 ▸). For instance, the cobalt oxalate (ox) bpmp phase {[Co(ox)(bpmp)]^.^3H_2_O}_*n*_, manifests a threefold inter­penetrated tri-periodic diamondoid (**dia**) topology. Use of the di­carboxyl­ate ligand oxy(bis­)benzoate (oba) afforded {[Co_3_(oba)_3_(bpmp)_2_]_*n*_, which exhibits a striking self-penetrated tri-periodic network with 4^4^5^17^6^7^ topology (Martin *et al.*, 2008 ▸). The title compound was isolated during an attempt to prepare a divalent nickel coordination polymer containing both bpmp and 4-(carboxyl­atoeth­yl)benzoato (ceb) ligands.

**Figure 1:**
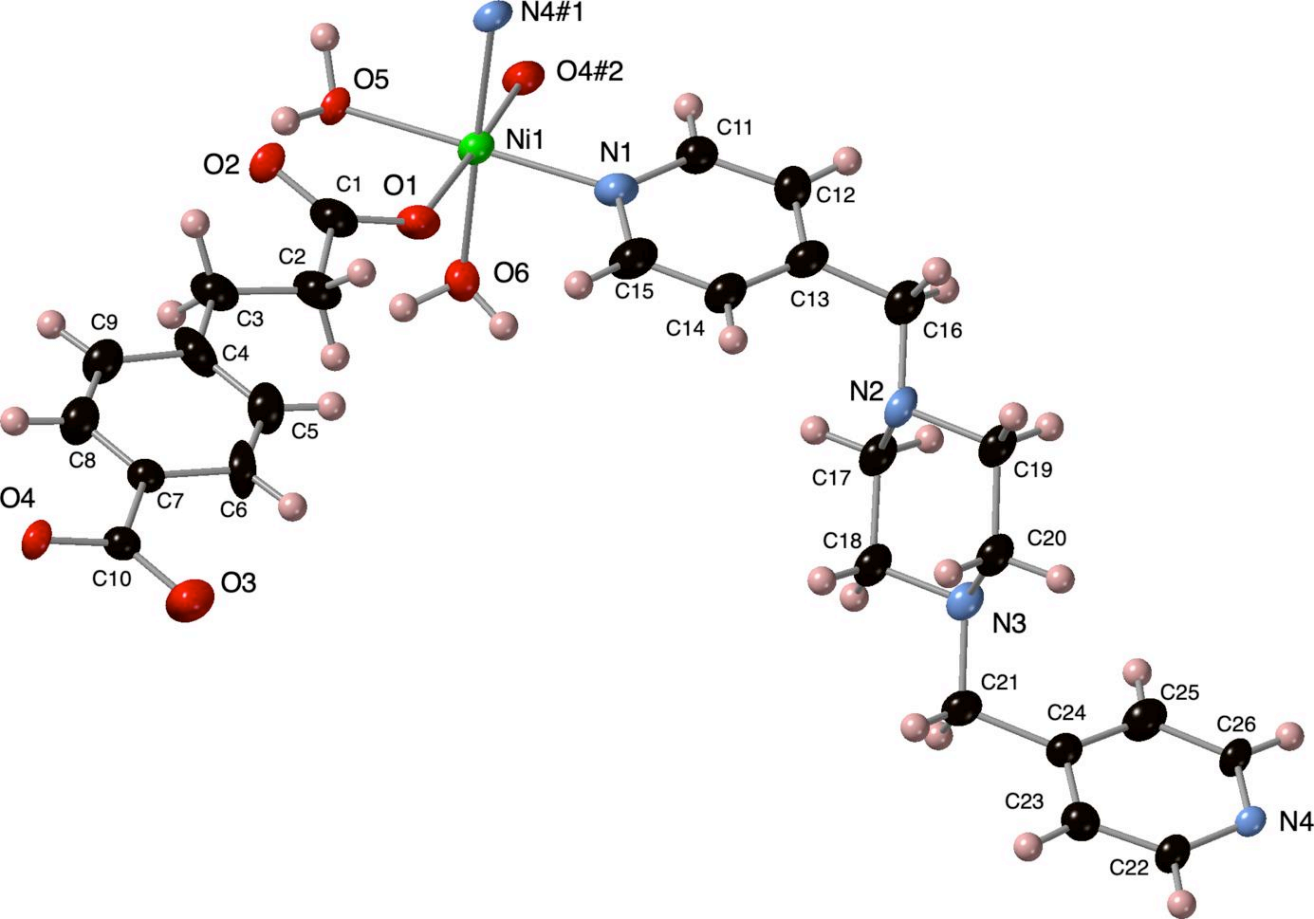
Nickel coordination environment in the title compound with full ceb and bpmp ligands. Displacement ellipsoids are drawn at the 50% probability level. Color code: Co, dark blue; O, red; N, light blue; C, black; H, pink. Symmetry codes are as listed in Table 1 ▸.

The asymmetric unit of the title compound contains a divalent nickel atom, a fully deprotonated ceb ligand, two bound water mol­ecules, and a bpmp ligand. The nickel atom is coordinated in an [O_4_N_2_] distorted octa­hedral fashion (Fig. 1 ▸) with two *cis*-oriented aqua ligands, two *trans*-oriented carboxyl­ate O atom donors from two ceb ligands, and two *cis*-oriented pyridyl N atom donors from two bpmp ligands. Pertinent bond length and angle information for the coordination sphere is listed in Table 1 ▸.

**Figure 2:**
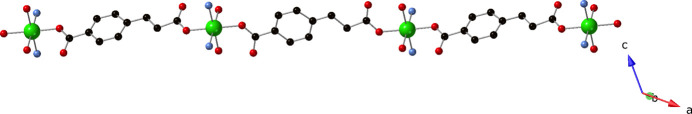
[Ni(ceb)(H_2_O)_2_]_*n*_ coordination polymer chain in the title compound.

**Figure 3:**
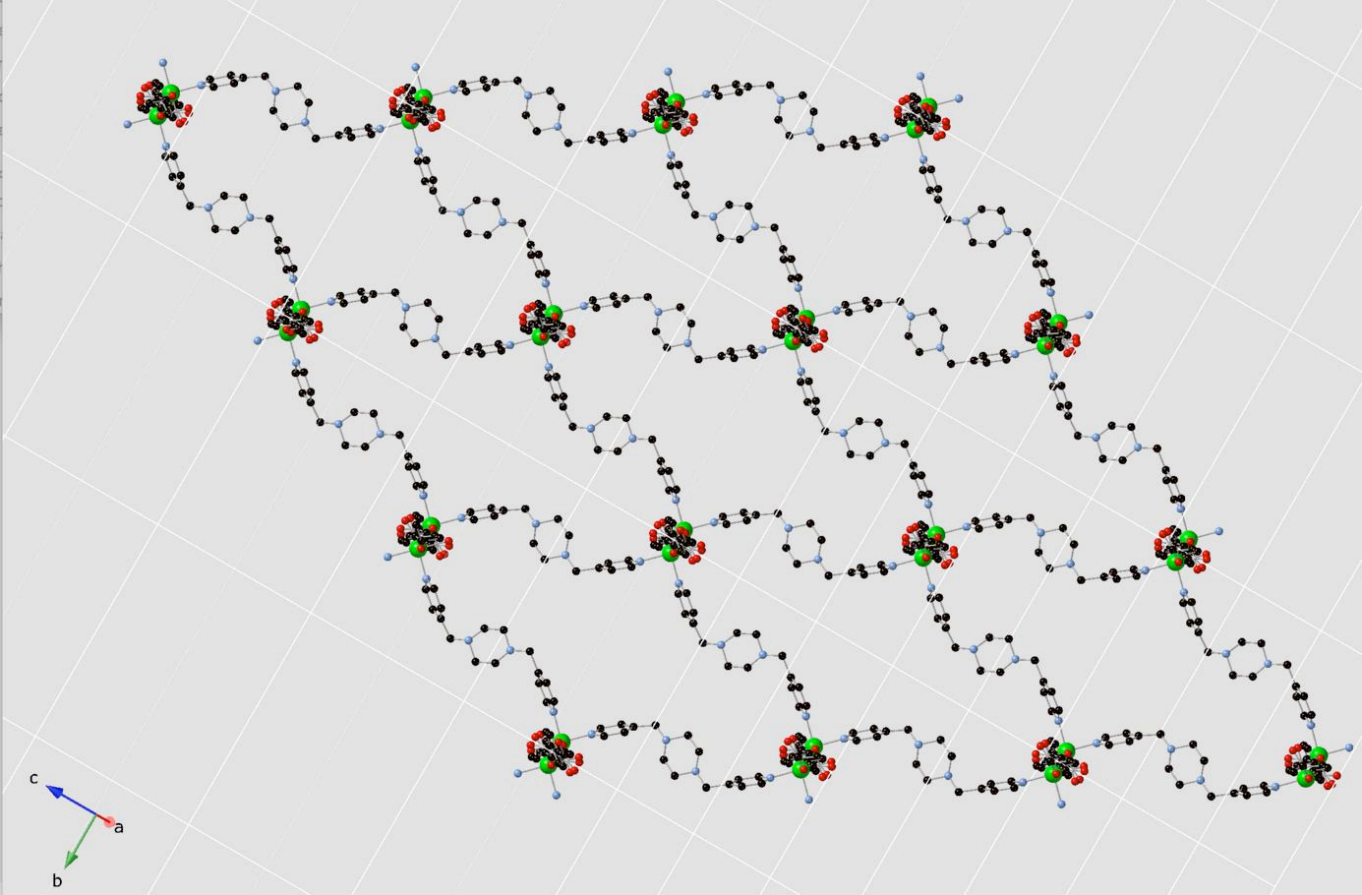
A single [Ni(ceb)(bpmp)(H_2_O)_2_]_*n*_ **dia** coordination polymer network in the title compound with unit cell outlines shown.

**Figure 4:**
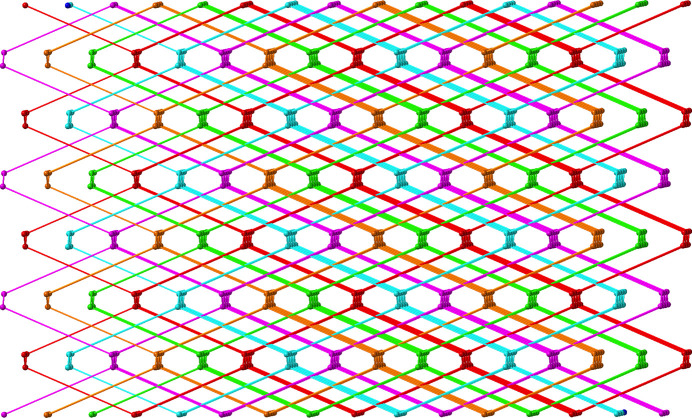
Fivefold inter­penetration of **dia** polymer networks in the title compound. The linking ligands are shown as rods.

The ceb ligands bridge adjacent nickel(II) atoms in a bis­(monodentate) fashion to construct [Ni(ceb)(H_2_O)_2_]_*n*_ chain submotifs arranged parallel to [201], in which the Ni⋯Ni inter­nuclear distance measures 13.996 (4) Å (Fig. 2 ▸). These chain motifs are connected into [Ni(ceb)(bpmp)(H_2_O)_2_]_n_ 6^6^ **dia** topology (Blatov *et al.*, 2014 ▸) coordination polymer networks (Fig. 3 ▸). Incipient void space within a single [Ni(ceb)(bpmp)(H_2_O)_2_]_*n*_ network allows inter­penetration of four additional networks to instill a fivefold system of inter­penetrated **dia** networks in the title compound (Fig. 4 ▸).

The [Ni(ceb)(H_2_O)_2_]_*n*_ chain submotifs are stabilized by inter­nal O—H⋯O hydrogen bonding between the bound water mol­ecules and unligated ceb carboxyl­ate O atoms (O5—H5*A*⋯O2). Adjacent [Ni(ceb)(bpmp)(H_2_O)_2_]_*n*_ coordination polymer networks are held into the fivefold inter­penetrated structure by similar O—H⋯O hydrogen bonding patterns between the bound water mol­ecules (O5, O6) and unligated ceb carboxyl­ate O atoms (O2^iv^, O3^iii^). Numerical details regarding the hydrogen bonding in the title compound are listed in Table 2 ▸.

## Synthesis and crystallization

Ni(NO_3_)_2_^.^6H_2_O (108 mg, 0.37 mmol), 4-(carb­oxy­eth­yl)benzoic acid (cebH_2_) (72 mg, 0.37 mmol), 1,4-bis­(pyridin-4-ylmeth­yl)piperazine (bpmp) (99 mg, 0.37 mmol), and 0.75 ml of a 1.0 *M* NaOH solution were placed into 10 ml distilled water in a Teflon-lined acid digestion bomb. The bomb was sealed and heated in an oven at 393 K for 48 h, and then cooled slowly to 273 K. Green crystals of the title complex were obtained in 72% yield.

## Refinement

Crystal data, data collection and structure refinement details are summarized in Table 3 ▸. The crystal was twinned by non-merohedry. Only data from the major twin component was used in solution and refinement. Additionally, the structure refined best as an inversion twin in space group *Cc* with a refined BASF parameter of 0.39 (4). The H atoms bound to the O atoms of the water mol­ecules were found by difference-Fourier maps, restrained with *DFIX* commands at 0.84 (2) Å, and refined with *U*_iso_(H) = 1.2*U*_eq_(O).

## Supplementary Material

Crystal structure: contains datablock(s) I, 1R. DOI: 10.1107/S2414314623007885/wm4194sup1.cif

Structure factors: contains datablock(s) I. DOI: 10.1107/S2414314623007885/wm4194Isup2.hkl

CCDC reference: 1946427

Additional supporting information:  crystallographic information; 3D view; checkCIF report

## Figures and Tables

**Table 1:** Selected geometric parameters (Å, °)

Ni1—O1	2.068 (10)	Ni1—O6	2.089 (11)
Ni1—O4^i^	2.071 (10)	Ni1—N1	2.089 (12)
Ni1—O5	2.112 (9)	Ni1—N4^ii^	2.109 (10)
			
O1—Ni1—O4^i^	174.77 (15)	O4^i^—Ni1—N4^ii^	89.8 (4)
O1—Ni1—O5	90.3 (4)	O6—Ni1—O5	90.47 (17)
O1—Ni1—O6	85.0 (4)	O6—Ni1—N4^ii^	178.3 (4)
O1—Ni1—N1	89.5 (4)	N1—Ni1—O5	179.7 (5)
O1—Ni1—N4^ii^	93.6 (4)	N1—Ni1—O6	89.3 (4)
O4^i^—Ni1—O5	85.8 (4)	N1—Ni1—N4^ii^	89.56 (16)
O4^i^—Ni1—O6	91.6 (3)	N4^ii^—Ni1—O5	90.6 (4)
O4^i^—Ni1—N1	94.4 (4)		

Symmetry codes: (i) 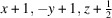 ; (ii) 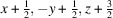 .

**Table 2:** Hydrogen-bond geometry (Å, °)

*D*—H⋯*A*	*D*—H	H⋯*A*	*D*⋯*A*	*D*—H⋯*A*
O5—H5*A*⋯O2	0.84 (3)	1.82 (4)	2.621 (15)	158 (9)
O5—H5*B*⋯O3^iii^	0.83 (3)	2.30 (7)	2.894 (14)	129 (6)
O6—H6*B*⋯O2^iv^	0.85 (3)	2.05 (4)	2.880 (15)	166 (9)

Symmetry codes: (iii) 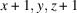 ; (iv) 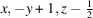 .

**Table 3:** Experimental details

Crystal data	
Chemical formula	[Ni(C_10_H_8_O_4_)(C_16_H_20_N_4_)(H_2_O)_2_]
*M*_r_	555.26
Crystal system, space group	Monoclinic, *C* *c*
Temperature (K)	173
*a*, *b*, *c* (Å)	17.282 (3), 16.324 (3), 12.698 (4)
β (°)	131.754 (2)
*V* (Å^3^)	2672.3 (11)
*Z*	4
Radiation type	Mo *K*α
μ (mm^−1^)	0.77
Crystal size (mm)	0.32 × 0.21 × 0.12

Data collection	
Diffractometer	Bruker APEXII CCD
Absorption correction	Multi-scan (*SADABS*; Krause *et al.*, 2015 ▸)
*T*_min_, *T*_max_	0.519, 0.745
No. of measured, independent and observed [*I* > 2σ(*I*)] reflections	21011, 4903, 3946
*R*_int_	0.118
(sin θ/λ)_max_ (Å^−1^)	0.604

Refinement	
*R*[*F* ^2^ > 2σ(*F* ^2^)], *wR*(*F* ^2^), *S*	0.057, 0.149, 1.05
No. of reflections	4903
No. of parameters	311
No. of restraints	8
H-atom treatment	H atoms treated by a mixture of independent and constrained refinement
Δρ_max_, Δρ_min_ (e Å^−3^)	1.46, −0.65
Absolute structure	Refined as an inversion twin
Absolute structure parameter	0.39 (4)

Computer programs: *COSMO* (Bruker, 2009 ▸), *SAINT* (Bruker, 2014 ▸), *SHELXT* (Sheldrick, 2015*a* ▸), *SHELXL* (Sheldrick, 2015*b* ▸), *CrystalMaker X* (Palmer, 2020 ▸), and *OLEX2* (Dolomanov *et al.*, 2009 ▸).

## full crystallographic data

### Crystal data

**Table:**

[Ni(C_10_H_8_O_4_)(C_16_H_20_N_4_)(H_2_O)_2_]	*F*(000) = 1168
*M**_r_* = 555.26	*D*_x_ = 1.380 Mg m^−^^3^
Monoclinic, *C**c*	Mo *K*α radiation, λ = 0.71073 Å
*a* = 17.282 (3) Å	Cell parameters from 7345 reflections
*b* = 16.324 (3) Å	θ = 2.5–25.1°
*c* = 12.698 (4) Å	µ = 0.77 mm^−^^1^
β = 131.754 (2)°	*T* = 173 K
*V* = 2672.3 (11) Å^3^	Chunk, green
*Z* = 4	0.32 × 0.21 × 0.12 mm

### Data collection

**Table:**

Bruker APEXII CCD diffractometer	4903 independent reflections
Radiation source: sealed tube	3946 reflections with *I* > 2σ(*I*)
Graphite monochromator	*R*_int_ = 0.118
Detector resolution: 8.36 pixels mm^-1^	θ_max_ = 25.4°, θ_min_ = 2.0°
ω scans	*h* = −20→20
Absorption correction: multi-scan (*SADABS*; Krause *et al.*, 2015)	*k* = −19→19
*T*_min_ = 0.519, *T*_max_ = 0.745	*l* = −15→15
21011 measured reflections	

### Refinement

**Table:**

Refinement on *F*^2^	Hydrogen site location: mixed
Least-squares matrix: full	H atoms treated by a mixture of independent and constrained refinement
*R*[*F*^2^ > 2σ(*F*^2^)] = 0.057	*w* = 1/[σ^2^(*F*_o_^2^) + (0.0734*P*)^2^ + 2.7583*P*] where *P* = (*F*_o_^2^ + 2*F*_c_^2^)/3
*wR*(*F*^2^) = 0.149	(Δ/σ)_max_ < 0.001
*S* = 1.05	Δρ_max_ = 1.46 e Å^−^^3^
4903 reflections	Δρ_min_ = −0.64 e Å^−^^3^
311 parameters	Absolute structure: Refined as an inversion twin
8 restraints	Absolute structure parameter: 0.39 (4)
Primary atom site location: dual	

### Special details

**Table:**

Experimental. Data was collected using a BRUKER CCD (charge coupled device) based diffractometer equipped with an Oxford low-temperature apparatus operating at 173 K. A suitable crystal was chosen and mounted on a nylon loop using Paratone oil. Data were measured using omega scans of 0.5° per frame for 30 s. The total number of images were based on results from the program COSMO where redundancy was expected to be 4 and completeness to 0.83Å to 100%. Cell parameters were retrieved using APEX II software and refined using SAINT on all observed reflections.Data reduction was performed using the SAINT software which corrects for Lp. Scaling and absorption corrections were applied using SADABS6 multi-scan technique, supplied by George Sheldrick. The structure was solved by the direct method using the SHELXT program and refined by least squares method on F2, SHELXL, incorporated in OLEX2.
Geometry. All esds (except the esd in the dihedral angle between two l.s. planes) are estimated using the full covariance matrix. The cell esds are taken into account individually in the estimation of esds in distances, angles and torsion angles; correlations between esds in cell parameters are only used when they are defined by crystal symmetry. An approximate (isotropic) treatment of cell esds is used for estimating esds involving l.s. planes.
Refinement. The structure was refined by Least Squares using version 2018/3 of XL (Sheldrick, 2015) incorporated in Olex2 (Dolomanov *et al.*, 2009). All non-hydrogen atoms were refined anisotropically. Hydrogen atom positions were calculated geometrically and refined using the riding model, except for the Hydrogen atom on the nitrogen atom which was found by difference Fourier methods and refined isotropically.

### Fractional atomic coordinates and isotropic or equivalent isotropic displacement parameters (Å^2^)

**Table:**

	*x*	*y*	*z*	*U*_iso_*/*U*_eq_	
Ni1	0.9080 (2)	0.44647 (4)	1.1095 (3)	0.0249 (3)	
O1	0.7548 (7)	0.4520 (6)	1.0183 (9)	0.031 (3)	
O2	0.7660 (7)	0.5196 (6)	1.1810 (9)	0.039 (3)	
O3	0.0511 (7)	0.4808 (7)	0.5374 (10)	0.042 (3)	
O4	0.0617 (7)	0.5475 (5)	0.7020 (8)	0.028 (3)	
O5	0.9473 (7)	0.5383 (6)	1.2552 (9)	0.029 (2)	
O6	0.8654 (7)	0.5358 (7)	0.9613 (10)	0.035 (3)	
N1	0.8685 (8)	0.3559 (7)	0.9646 (11)	0.026 (3)	
N2	0.7051 (9)	0.2250 (7)	0.5017 (11)	0.030 (3)	
N3	0.6112 (9)	0.2750 (7)	0.2218 (11)	0.034 (3)	
N4	0.4467 (8)	0.1455 (7)	−0.2456 (11)	0.025 (3)	
C1	0.7170 (12)	0.4855 (9)	1.0645 (17)	0.035 (2)	
C2	0.5985 (10)	0.4809 (8)	0.9613 (13)	0.035 (2)	
H2A	0.570123	0.503193	0.869034	0.042*	
H2B	0.577580	0.422672	0.946978	0.042*	
C3	0.5517 (11)	0.5259 (8)	1.0082 (14)	0.035 (2)	
H3A	0.586039	0.507320	1.104771	0.042*	
H3B	0.568175	0.584794	1.014318	0.042*	
C4	0.4387 (11)	0.5181 (9)	0.9203 (18)	0.039 (4)	
C5	0.3753 (13)	0.4740 (9)	0.7964 (17)	0.043 (4)	
H5	0.405671	0.444413	0.767356	0.051*	
C6	0.2662 (11)	0.4715 (8)	0.7110 (15)	0.041 (3)	
H6	0.224770	0.440385	0.626232	0.049*	
C7	0.2181 (8)	0.5153 (8)	0.7515 (12)	0.0261 (18)	
C8	0.2775 (7)	0.5557 (5)	0.8700 (9)	0.042 (2)	
H8	0.247516	0.583759	0.900809	0.050*	
C9	0.3845 (7)	0.5582 (5)	0.9521 (9)	0.0390 (19)	
H9	0.424024	0.589608	1.036478	0.047*	
C10	0.1021 (10)	0.5149 (9)	0.6574 (14)	0.0261 (18)	
C11	0.9436 (10)	0.3177 (9)	0.9745 (14)	0.034 (4)	
H11	1.014340	0.329718	1.052678	0.041*	
C12	0.9193 (12)	0.2603 (9)	0.8714 (15)	0.037 (4)	
H12	0.973385	0.233259	0.883407	0.045*	
C13	0.8190 (11)	0.2445 (10)	0.7565 (15)	0.034 (4)	
C14	0.7419 (11)	0.2820 (9)	0.7461 (15)	0.034 (4)	
H14	0.670952	0.269867	0.669043	0.041*	
C15	0.7697 (11)	0.3374 (10)	0.8497 (14)	0.039 (4)	
H15	0.715707	0.363767	0.838687	0.046*	
C16	0.7929 (12)	0.1936 (10)	0.6390 (14)	0.037 (4)	
H16A	0.853783	0.191376	0.646851	0.044*	
H16B	0.777492	0.137020	0.648371	0.044*	
C17	0.7384 (12)	0.2994 (9)	0.4741 (15)	0.0340 (7)	
H17A	0.795601	0.285564	0.477098	0.041*	
H17B	0.763878	0.341043	0.547575	0.041*	
C18	0.6462 (11)	0.3335 (8)	0.3286 (13)	0.0340 (7)	
H18A	0.589034	0.347020	0.325883	0.041*	
H18B	0.667120	0.384459	0.310988	0.041*	
C19	0.6673 (11)	0.1634 (8)	0.3875 (13)	0.0340 (7)	
H19A	0.725343	0.147538	0.393179	0.041*	
H19B	0.642672	0.113524	0.401786	0.041*	
C20	0.5811 (12)	0.1987 (8)	0.2451 (15)	0.0340 (7)	
H20A	0.560144	0.158647	0.171595	0.041*	
H20B	0.520475	0.208814	0.236221	0.041*	
C21	0.5270 (12)	0.3104 (9)	0.0812 (14)	0.036 (4)	
H21A	0.549806	0.363581	0.072359	0.044*	
H21B	0.465634	0.320556	0.070690	0.044*	
C22	0.3756 (11)	0.1827 (9)	−0.2542 (13)	0.031 (3)	
H22	0.305146	0.173130	−0.336325	0.037*	
C23	0.3944 (11)	0.2335 (8)	−0.1559 (15)	0.032 (3)	
H23	0.338597	0.256557	−0.167464	0.039*	
C24	0.4971 (11)	0.2519 (9)	−0.0363 (14)	0.029 (3)	
C25	0.5714 (12)	0.2170 (10)	−0.0316 (16)	0.041 (4)	
H25	0.642522	0.228510	0.045559	0.050*	
C26	0.5443 (10)	0.1653 (8)	−0.1371 (12)	0.030 (3)	
H26	0.597494	0.143039	−0.131876	0.036*	
H5A	0.898 (4)	0.537 (6)	1.254 (8)	0.036*	
H6A	0.928 (3)	0.545 (6)	1.007 (7)	0.036*	
H5B	1.000 (4)	0.514 (5)	1.323 (6)	0.036*	
H6B	0.830 (5)	0.527 (5)	0.874 (4)	0.036*	

### Atomic displacement parameters (Å^2^)

**Table:**

	*U*^11^	*U*^22^	*U*^33^	*U*^12^	*U*^13^	*U*^23^
Ni1	0.0212 (4)	0.0340 (4)	0.0202 (4)	−0.0002 (11)	0.0140 (3)	−0.0002 (11)
O1	0.008 (4)	0.049 (7)	0.025 (6)	0.003 (4)	0.007 (4)	0.002 (5)
O2	0.032 (5)	0.055 (7)	0.021 (6)	0.009 (5)	0.013 (4)	−0.006 (5)
O3	0.025 (5)	0.074 (8)	0.035 (6)	0.003 (5)	0.024 (5)	−0.002 (6)
O4	0.032 (5)	0.036 (6)	0.024 (6)	0.002 (4)	0.022 (5)	−0.006 (4)
O5	0.020 (5)	0.039 (5)	0.019 (5)	0.000 (4)	0.010 (4)	−0.010 (4)
O6	0.030 (6)	0.046 (6)	0.036 (6)	0.005 (5)	0.025 (5)	−0.001 (5)
N1	0.019 (6)	0.035 (6)	0.024 (6)	−0.001 (5)	0.014 (5)	0.007 (5)
N2	0.043 (7)	0.025 (6)	0.027 (7)	−0.010 (5)	0.026 (6)	−0.009 (5)
N3	0.042 (8)	0.035 (7)	0.026 (7)	0.002 (6)	0.024 (6)	0.000 (5)
N4	0.028 (6)	0.029 (6)	0.017 (6)	0.004 (5)	0.015 (5)	−0.001 (5)
C1	0.034 (4)	0.030 (4)	0.044 (5)	0.004 (3)	0.027 (4)	0.014 (3)
C2	0.034 (4)	0.030 (4)	0.044 (5)	0.004 (3)	0.027 (4)	0.014 (3)
C3	0.034 (4)	0.030 (4)	0.044 (5)	0.004 (3)	0.027 (4)	0.014 (3)
C4	0.033 (7)	0.028 (7)	0.068 (9)	0.008 (6)	0.039 (7)	0.011 (7)
C5	0.060 (9)	0.034 (7)	0.054 (9)	0.002 (7)	0.046 (8)	−0.005 (7)
C6	0.026 (5)	0.044 (6)	0.054 (7)	−0.008 (4)	0.027 (5)	−0.030 (5)
C7	0.016 (4)	0.034 (4)	0.025 (4)	0.003 (3)	0.012 (3)	0.002 (3)
C8	0.035 (4)	0.055 (5)	0.039 (5)	−0.001 (4)	0.026 (4)	−0.011 (4)
C9	0.034 (4)	0.049 (5)	0.032 (4)	−0.002 (3)	0.021 (4)	−0.008 (4)
C10	0.016 (4)	0.034 (4)	0.025 (4)	0.003 (3)	0.012 (3)	0.002 (3)
C11	0.021 (7)	0.052 (9)	0.028 (9)	0.000 (7)	0.016 (7)	−0.003 (7)
C12	0.044 (9)	0.038 (8)	0.039 (9)	0.004 (6)	0.032 (8)	−0.001 (7)
C13	0.040 (9)	0.038 (8)	0.025 (9)	−0.005 (7)	0.022 (7)	0.001 (6)
C14	0.031 (8)	0.047 (9)	0.024 (8)	−0.005 (7)	0.018 (7)	−0.007 (7)
C15	0.026 (8)	0.062 (10)	0.030 (8)	−0.005 (7)	0.019 (7)	−0.002 (7)
C16	0.044 (9)	0.035 (8)	0.033 (9)	−0.006 (7)	0.026 (7)	−0.008 (6)
C17	0.0454 (17)	0.0312 (15)	0.0290 (14)	−0.0073 (12)	0.0263 (14)	−0.0071 (12)
C18	0.0454 (17)	0.0312 (15)	0.0290 (14)	−0.0073 (12)	0.0263 (14)	−0.0071 (12)
C19	0.0454 (17)	0.0312 (15)	0.0290 (14)	−0.0073 (12)	0.0263 (14)	−0.0071 (12)
C20	0.0454 (17)	0.0312 (15)	0.0290 (14)	−0.0073 (12)	0.0263 (14)	−0.0071 (12)
C21	0.050 (9)	0.033 (8)	0.028 (8)	0.005 (7)	0.027 (7)	0.006 (6)
C22	0.026 (7)	0.037 (8)	0.024 (8)	0.003 (7)	0.014 (7)	−0.003 (7)
C23	0.024 (7)	0.037 (7)	0.031 (8)	0.002 (6)	0.017 (6)	−0.002 (6)
C24	0.039 (9)	0.025 (7)	0.025 (8)	0.004 (6)	0.022 (7)	0.004 (6)
C25	0.029 (8)	0.052 (10)	0.028 (9)	−0.005 (7)	0.013 (7)	−0.007 (7)
C26	0.023 (7)	0.045 (8)	0.021 (7)	0.004 (6)	0.014 (6)	−0.006 (6)

### Geometric parameters (Å, º)

**Table:**

Ni1—O1	2.068 (10)	C7—C8	1.302 (14)
Ni1—O4^i^	2.071 (10)	C7—C10	1.501 (17)
Ni1—O5	2.112 (9)	C8—H8	0.9500
Ni1—O6	2.089 (11)	C8—C9	1.393 (11)
Ni1—N1	2.089 (12)	C9—H9	0.9500
Ni1—N4^ii^	2.109 (10)	C11—H11	0.9500
O1—C1	1.258 (18)	C11—C12	1.43 (2)
O2—C1	1.243 (18)	C12—H12	0.9500
O3—C10	1.272 (16)	C12—C13	1.35 (2)
O4—C10	1.273 (16)	C13—C14	1.39 (2)
O5—H5A	0.84 (3)	C13—C16	1.490 (19)
O5—H5B	0.83 (3)	C14—H14	0.9500
O6—H6A	0.83 (3)	C14—C15	1.39 (2)
O6—H6B	0.85 (3)	C15—H15	0.9500
N1—C11	1.368 (18)	C16—H16A	0.9900
N1—C15	1.349 (17)	C16—H16B	0.9900
N2—C16	1.442 (17)	C17—H17A	0.9900
N2—C17	1.482 (18)	C17—H17B	0.9900
N2—C19	1.510 (15)	C17—C18	1.530 (19)
N3—C18	1.423 (15)	C18—H18A	0.9900
N3—C20	1.454 (17)	C18—H18B	0.9900
N3—C21	1.476 (16)	C19—H19A	0.9900
N4—C22	1.309 (18)	C19—H19B	0.9900
N4—C26	1.323 (16)	C19—C20	1.495 (19)
C1—C2	1.531 (19)	C20—H20A	0.9900
C2—H2A	0.9900	C20—H20B	0.9900
C2—H2B	0.9900	C21—H21A	0.9900
C2—C3	1.479 (19)	C21—H21B	0.9900
C3—H3A	0.9900	C21—C24	1.543 (19)
C3—H3B	0.9900	C22—H22	0.9500
C3—C4	1.47 (2)	C22—C23	1.347 (19)
C4—C5	1.377 (10)	C23—H23	0.9500
C4—C9	1.404 (17)	C23—C24	1.40 (2)
C5—H5	0.9500	C24—C25	1.37 (2)
C5—C6	1.42 (2)	C25—H25	0.9500
C6—H6	0.9500	C25—C26	1.37 (2)
C6—C7	1.427 (16)	C26—H26	0.9500
			
O1—Ni1—O4^i^	174.77 (15)	O3—C10—C7	116.7 (12)
O1—Ni1—O5	90.3 (4)	O4—C10—C7	118.8 (12)
O1—Ni1—O6	85.0 (4)	N1—C11—H11	118.9
O1—Ni1—N1	89.5 (4)	N1—C11—C12	122.3 (13)
O1—Ni1—N4^ii^	93.6 (4)	C12—C11—H11	118.9
O4^i^—Ni1—O5	85.8 (4)	C11—C12—H12	120.1
O4^i^—Ni1—O6	91.6 (3)	C13—C12—C11	119.9 (15)
O4^i^—Ni1—N1	94.4 (4)	C13—C12—H12	120.1
O4^i^—Ni1—N4^ii^	89.8 (4)	C12—C13—C14	118.4 (15)
O6—Ni1—O5	90.47 (17)	C12—C13—C16	120.2 (15)
O6—Ni1—N4^ii^	178.3 (4)	C14—C13—C16	121.2 (13)
N1—Ni1—O5	179.7 (5)	C13—C14—H14	120.3
N1—Ni1—O6	89.3 (4)	C13—C14—C15	119.4 (14)
N1—Ni1—N4^ii^	89.56 (16)	C15—C14—H14	120.3
N4^ii^—Ni1—O5	90.6 (4)	N1—C15—C14	124.0 (15)
C1—O1—Ni1	128.8 (10)	N1—C15—H15	118.0
C10—O4—Ni1^iii^	130.1 (9)	C14—C15—H15	118.0
Ni1—O5—H5A	103 (7)	N2—C16—C13	112.7 (13)
Ni1—O5—H5B	93 (6)	N2—C16—H16A	109.1
H5A—O5—H5B	114 (6)	N2—C16—H16B	109.1
Ni1—O6—H6A	89 (6)	C13—C16—H16A	109.1
Ni1—O6—H6B	125 (6)	C13—C16—H16B	109.1
H6A—O6—H6B	115 (6)	H16A—C16—H16B	107.8
C11—N1—Ni1	120.6 (9)	N2—C17—H17A	109.8
C15—N1—Ni1	123.2 (11)	N2—C17—H17B	109.8
C15—N1—C11	115.9 (13)	N2—C17—C18	109.2 (12)
C16—N2—C17	108.4 (12)	H17A—C17—H17B	108.3
C16—N2—C19	111.0 (11)	C18—C17—H17A	109.8
C17—N2—C19	107.8 (10)	C18—C17—H17B	109.8
C18—N3—C20	111.3 (10)	N3—C18—C17	110.2 (11)
C18—N3—C21	109.9 (12)	N3—C18—H18A	109.6
C20—N3—C21	112.5 (12)	N3—C18—H18B	109.6
C22—N4—Ni1^iv^	121.5 (9)	C17—C18—H18A	109.6
C22—N4—C26	116.6 (12)	C17—C18—H18B	109.6
C26—N4—Ni1^iv^	121.8 (10)	H18A—C18—H18B	108.1
O1—C1—C2	113.2 (14)	N2—C19—H19A	109.6
O2—C1—O1	126.6 (14)	N2—C19—H19B	109.6
O2—C1—C2	120.2 (14)	H19A—C19—H19B	108.1
C1—C2—H2A	108.7	C20—C19—N2	110.5 (11)
C1—C2—H2B	108.7	C20—C19—H19A	109.6
H2A—C2—H2B	107.6	C20—C19—H19B	109.6
C3—C2—C1	114.1 (12)	N3—C20—C19	111.9 (13)
C3—C2—H2A	108.7	N3—C20—H20A	109.2
C3—C2—H2B	108.7	N3—C20—H20B	109.2
C2—C3—H3A	108.0	C19—C20—H20A	109.2
C2—C3—H3B	108.0	C19—C20—H20B	109.2
H3A—C3—H3B	107.3	H20A—C20—H20B	107.9
C4—C3—C2	117.1 (13)	N3—C21—H21A	109.5
C4—C3—H3A	108.0	N3—C21—H21B	109.5
C4—C3—H3B	108.0	N3—C21—C24	110.6 (12)
C5—C4—C3	123.7 (10)	H21A—C21—H21B	108.1
C5—C4—C9	113.4 (8)	C24—C21—H21A	109.5
C9—C4—C3	122.9 (14)	C24—C21—H21B	109.5
C4—C5—H5	119.0	N4—C22—H22	117.4
C4—C5—C6	122.0 (10)	N4—C22—C23	125.1 (13)
C6—C5—H5	119.0	C23—C22—H22	117.4
C5—C6—H6	119.8	C22—C23—H23	120.4
C5—C6—C7	120.5 (12)	C22—C23—C24	119.1 (14)
C7—C6—H6	119.8	C24—C23—H23	120.4
C6—C7—C10	120.0 (11)	C23—C24—C21	123.2 (14)
C8—C7—C6	118.0 (11)	C25—C24—C21	121.0 (13)
C8—C7—C10	122.0 (11)	C25—C24—C23	115.7 (14)
C7—C8—H8	119.6	C24—C25—H25	119.6
C7—C8—C9	120.8 (8)	C24—C25—C26	120.8 (14)
C9—C8—H8	119.6	C26—C25—H25	119.6
C4—C9—H9	117.4	N4—C26—C25	122.4 (14)
C8—C9—C4	125.3 (9)	N4—C26—H26	118.8
C8—C9—H9	117.4	C25—C26—H26	118.8
O3—C10—O4	124.6 (12)		
			
Ni1—O1—C1—O2	−4 (2)	C9—C4—C5—C6	0.7 (13)
Ni1—O1—C1—C2	176.3 (8)	C10—C7—C8—C9	−176.9 (11)
Ni1^iii^—O4—C10—O3	3 (2)	C11—N1—C15—C14	1 (2)
Ni1^iii^—O4—C10—C7	−176.3 (8)	C11—C12—C13—C14	−3 (2)
Ni1—N1—C11—C12	−174.9 (10)	C11—C12—C13—C16	171.6 (13)
Ni1—N1—C15—C14	174.7 (11)	C12—C13—C14—C15	3 (2)
Ni1^iv^—N4—C22—C23	170.4 (11)	C12—C13—C16—N2	−139.0 (15)
Ni1^iv^—N4—C26—C25	−171.1 (11)	C13—C14—C15—N1	−2 (2)
O1—C1—C2—C3	−175.1 (11)	C14—C13—C16—N2	36 (2)
O2—C1—C2—C3	5 (2)	C15—N1—C11—C12	−1 (2)
N1—C11—C12—C13	2 (2)	C16—N2—C17—C18	179.9 (11)
N2—C17—C18—N3	−61.5 (14)	C16—N2—C19—C20	−176.2 (13)
N2—C19—C20—N3	55.7 (15)	C16—C13—C14—C15	−171.7 (14)
N3—C21—C24—C23	134.8 (15)	C17—N2—C16—C13	73.5 (16)
N3—C21—C24—C25	−48 (2)	C17—N2—C19—C20	−57.6 (13)
N4—C22—C23—C24	3 (2)	C18—N3—C20—C19	−56.5 (14)
C1—C2—C3—C4	−174.7 (12)	C18—N3—C21—C24	173.1 (11)
C2—C3—C4—C5	−2.3 (16)	C19—N2—C16—C13	−168.2 (11)
C2—C3—C4—C9	−179.4 (11)	C19—N2—C17—C18	59.7 (13)
C3—C4—C5—C6	−176.6 (19)	C20—N3—C18—C17	58.4 (14)
C3—C4—C9—C8	177.3 (11)	C20—N3—C21—C24	−62.3 (16)
C4—C5—C6—C7	0 (2)	C21—N3—C18—C17	−176.2 (13)
C5—C4—C9—C8	−0.1 (13)	C21—N3—C20—C19	179.7 (11)
C5—C6—C7—C8	−2 (2)	C21—C24—C25—C26	−179.6 (14)
C5—C6—C7—C10	177.5 (14)	C22—N4—C26—C25	5 (2)
C6—C7—C8—C9	2.1 (17)	C22—C23—C24—C21	179.0 (13)
C6—C7—C10—O3	−6 (2)	C22—C23—C24—C25	1 (2)
C6—C7—C10—O4	173.1 (13)	C23—C24—C25—C26	−2 (2)
C7—C8—C9—C4	−1.4 (15)	C24—C25—C26—N4	−1 (3)
C8—C7—C10—O3	172.7 (12)	C26—N4—C22—C23	−6 (2)
C8—C7—C10—O4	−8 (2)		

Symmetry codes: (i) *x*+1, −*y*+1, *z*+1/2; (ii) *x*+1/2, −*y*+1/2, *z*+3/2; (iii) *x*−1, −*y*+1, *z*−1/2; (iv) *x*−1/2, −*y*+1/2, *z*−3/2.

### Hydrogen-bond geometry (Å, º)

**Table:**

*D*—H···*A*	*D*—H	H···*A*	*D*···*A*	*D*—H···*A*
O5—H5*A*···O2	0.84 (3)	1.82 (4)	2.621 (15)	158 (9)
O5—H5*B*···O3^v^	0.83 (3)	2.30 (7)	2.894 (14)	129 (6)
O6—H6*B*···O2^vi^	0.85 (3)	2.05 (4)	2.880 (15)	166 (9)

Symmetry codes: (v) *x*+1, *y*, *z*+1; (vi) *x*, −*y*+1, *z*−1/2.
